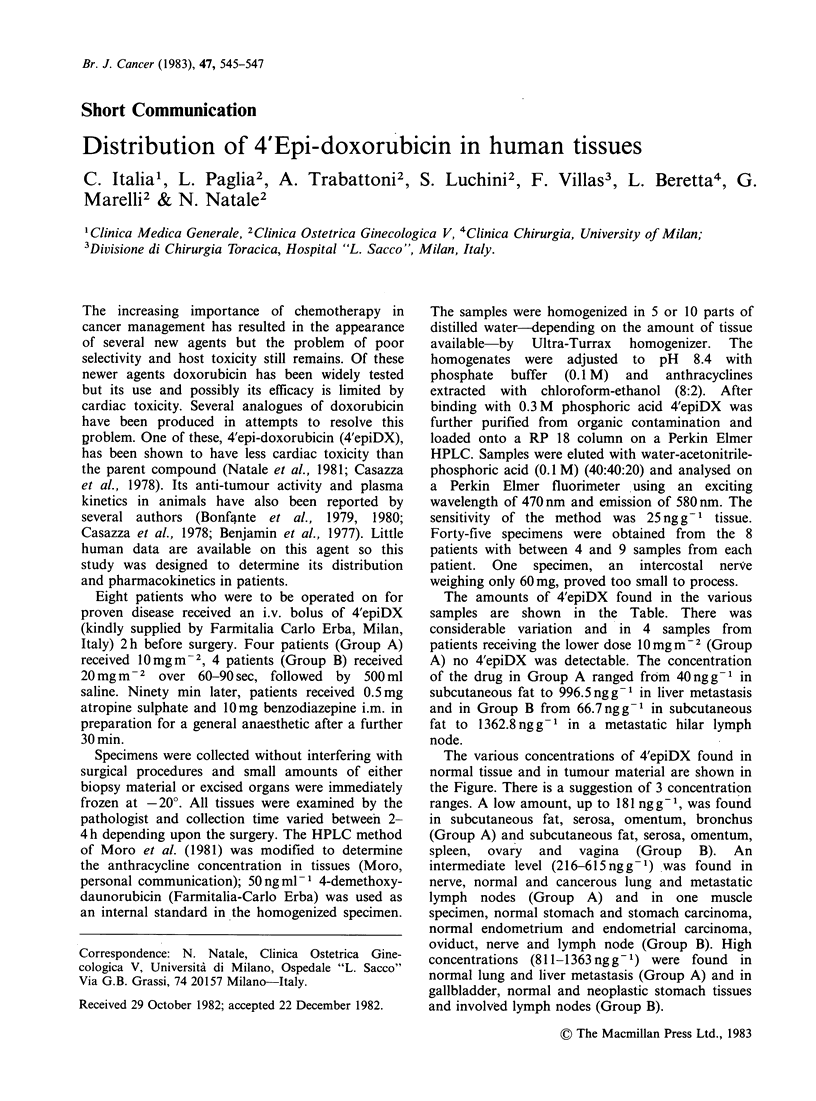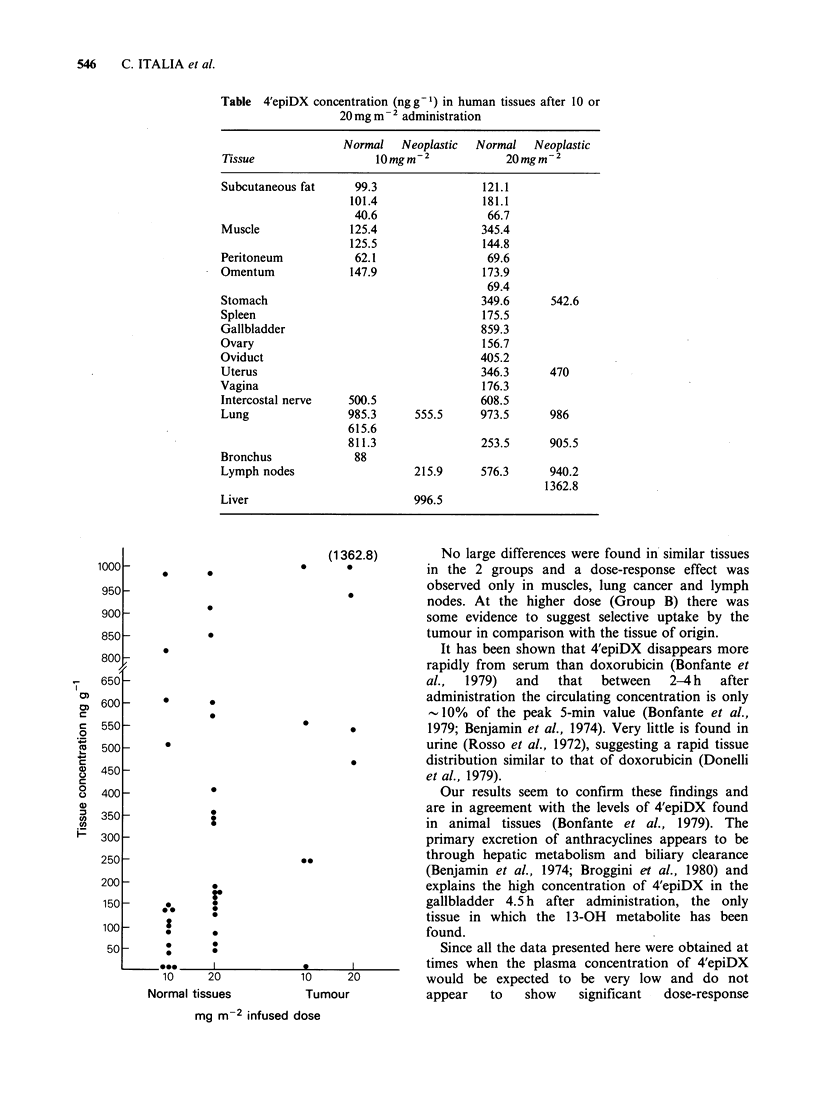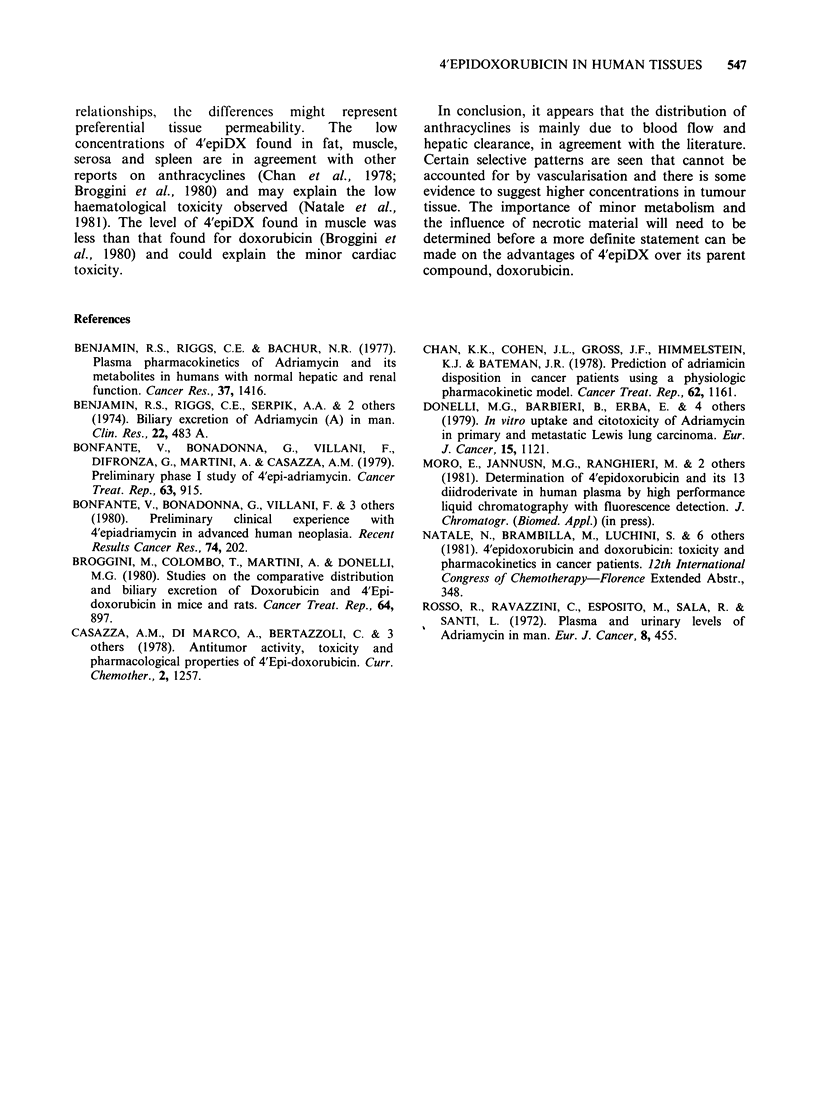# Distribution of 4'epi-doxorubicin in human tissues.

**DOI:** 10.1038/bjc.1983.86

**Published:** 1983-04

**Authors:** C. Italia, L. Paglia, A. Trabattoni, S. Luchini, F. Villas, L. Beretta, G. Marelli, N. Natale


*Br. J. Cancer* (1983), **47**, 545–547

**Short Communication**

### Distribution of 4′Epi-doxorubicin in human tissues

C. Italia[1], L. Paglia[2], A. Trabattoni[2], S. Luchini[2], F. Villas[3], L. Beretta[4], G. Marelli[2] & N. Natale[2]

[1]*Clinica Medica Generale,* [2]*Clinica Ostetrica Ginecologica V,* [4]*Clinica Chirurgia, University of Milan;* [3]*Divisione di Chirurgia Toracica, Hospital "L. Sacco", Milan, Italy.*

The increasing importance of chemotherapy in cancer management has resulted in the appearance of several new agents but the problem of poor selectivity and host toxicity still remains. Of these newer agents doxorubicin has been widely tested but its use and possibly its efficacy is limited by cardiac toxicity. Several analogues of doxorubicin have been produced in attempts to resolve this problem. One of these, 4′epi-doxorubicin (4′epiDX), has been shown to have less cardiac toxicity than the parent compound (Natale *et al.*, 1981; Casazza *et al.*, 1978). Its anti-tumour activity and plasma kinetics in animals have also been reported by several authors (Bonfante *et al.*, 1979, 1980; Casazza *et al.*, 1978; Benjamin *et al.*, 1977). Little human data are available on this agent so this study was designed to determine its distribution and pharmacokinetics in patients.

Eight patients who were to be operated on for proven disease received an i.v. bolus of 4′epiDX (kindly supplied by Farmitalia Carlo Erba, Milan, Italy) 2 h before surgery. Four patients (Group A) received $10\,mg\,m^{-2}$, 4 patients (Group B) received $20\,mg\,m^{-2}$ over 60–90 sec, followed by 500 ml saline. Ninety min later, patients received 0.5 mg atropine sulphate and 10 mg benzodiazepine i.m. in preparation for a general anaesthetic after a further 30 min.

Specimens were collected without interfering with surgical procedures and small amounts of either biopsy material or excised organs were immediately frozen at −20°. All tissues were examined by the pathologist and collection time varied between 2–4 h depending upon the surgery. The HPLC method of Moro *et al.* (1981) was modified to determine the anthracycline concentration in tissues (Moro, personal communication); $50\,ng\,ml^{-1}$ 4-demethoxy-daunorubicin (Farmitalia-Carlo Erba) was used as an internal standard in the homogenized specimen.

The samples were homogenized in 5 or 10 parts of distilled water—depending on the amount of tissue available—by Ultra-Turrax homogenizer. The homogenates were adjusted to pH 8.4 with phosphate buffer (0.1 M) and anthracyclines extracted with chloroform-ethanol (8:2). After binding with 0.3 M phosphoric acid 4′epiDX was further purified from organic contamination and loaded onto a RP 18 column on a Perkin Elmer HPLC. Samples were eluted with water-acetonitrile-phosphoric acid (0.1 M) (40:40:20) and analysed on a Perkin Elmer fluorimeter using an exciting wavelength of 470 nm and emission of 580 nm. The sensitivity of the method was $25\,ng\,g^{-1}$ tissue. Forty-five specimens were obtained from the 8 patients with between 4 and 9 samples from each patient. One specimen, an intercostal nerve weighing only 60 mg, proved too small to process.

The amounts of 4′epiDX found in the various samples are shown in the Table. There was considerable variation and in 4 samples from patients receiving the lower dose $10\,mg\,m^{-2}$ (Group A) no 4′epiDX was detectable. The concentration of the drug in Group A ranged from $40\,ng\,g^{-1}$ in subcutaneous fat to $996.5\,ng\,g^{-1}$ in liver metastasis and in Group B from $66.7\,ng\,g^{-1}$ in subcutaneous fat to $1362.8\,ng\,g^{-1}$ in a metastatic hilar lymph node.

The various concentrations of 4′epiDX found in normal tissue and in tumour material are shown in the Figure. There is a suggestion of 3 concentration ranges. A low amount, up to $181\,ng\,g^{-1}$, was found in subcutaneous fat, serosa, omentum, bronchus (Group A) and subcutaneous fat, serosa, omentum, spleen, ovary and vagina (Group B). An intermediate level ($216–615\,ng\,g^{-1}$) was found in nerve, normal and cancerous lung and metastatic lymph nodes (Group A) and in one muscle specimen, normal stomach and stomach carcinoma, normal endometrium and endometrial carcinoma, oviduct, nerve and lymph node (Group B). High concentrations ($811–1363\,ng\,g^{-1}$) were found in normal lung and liver metastasis (Group A) and in gallbladder, normal and neoplastic stomach tissues and involved lymph nodes (Group B).

Correspondence: N. Natale, Clinica Ostetrica Ginecologica V, Università di Milano, Ospedale "L. Sacco" Via G.B. Grassi, 74 20157 Milano—Italy.

Received 29 October 1982; accepted 22 December 1982.

© The Macmillan Press Ltd., 1983

**Table** 4′epiDX concentration (ng $g^{-1}$) in human tissues after 10 or 20 mg $m^{-2}$ administration

| *Tissue* | *Normal* 10 *mg* $m^{-2}$ | *Neoplastic* 10 *mg* $m^{-2}$ | *Normal* 20 *mg* $m^{-2}$ | *Neoplastic* 20 *mg* $m^{-2}$ |
|---|---|---|---|---|
| Subcutaneous fat | 99.3 | | 121.1 | |
| | 101.4 | | 181.1 | |
| | 40.6 | | 66.7 | |
| Muscle | 125.4 | | 345.4 | |
| | 125.5 | | 144.8 | |
| Peritoneum | 62.1 | | 69.6 | |
| Omentum | 147.9 | | 173.9 | |
| | | | 69.4 | |
| Stomach | | | 349.6 | 542.6 |
| Spleen | | | 175.5 | |
| Gallbladder | | | 859.3 | |
| Ovary | | | 156.7 | |
| Oviduct | | | 405.2 | |
| Uterus | | | 346.3 | 470 |
| Vagina | | | 176.3 | |
| Intercostal nerve | 500.5 | | 608.5 | |
| Lung | 985.3 | 555.5 | 973.5 | 986 |
| | 615.6 | | | |
| | 811.3 | | 253.5 | 905.5 |
| Bronchus | 88 | | | |
| Lymph nodes | | 215.9 | 576.3 | 940.2 |
| | | | | 1362.8 |
| Liver | | 996.5 | | |

No large differences were found in similar tissues in the 2 groups and a dose-response effect was observed only in muscles, lung cancer and lymph nodes. At the higher dose (Group B) there was some evidence to suggest selective uptake by the tumour in comparison with the tissue of origin.

It has been shown that 4′epiDX disappears more rapidly from serum than doxorubicin (Bonfante *et al.*, 1979) and that between 2–4 h after administration the circulating concentration is only ~10% of the peak 5-min value (Bonfante *et al.*, 1979; Benjamin *et al.*, 1974). Very little is found in urine (Rosso *et al.*, 1972), suggesting a rapid tissue distribution similar to that of doxorubicin (Donelli *et al.*, 1979).

Our results seem to confirm these findings and are in agreement with the levels of 4′epiDX found in animal tissues (Bonfante *et al.*, 1979). The primary excretion of anthracyclines appears to be through hepatic metabolism and biliary clearance (Benjamin *et al.*, 1974; Broggini *et al.*, 1980) and explains the high concentration of 4′epiDX in the gallbladder 4.5 h after administration, the only tissue in which the 13-OH metabolite has been found.

Since all the data presented here were obtained at times when the plasma concentration of 4′epiDX would be expected to be very low and do not appear to show significant dose-response relationships, thc differences might represent preferential tissue permeability. The low concentrations of 4'epiDX found in fat, muscle, serosa and spleen are in agreement with other reports on anthracyclines (Chan *et al.*, 1978; Broggini *et al.*, 1980) and may explain the low haematological toxicity observed (Natale *et al.*, 1981). The level of 4'epiDX found in muscle was less than that found for doxorubicin (Broggini *et al.*, 1980) and could explain the minor cardiac toxicity.

In conclusion, it appears that the distribution of anthracyclines is mainly due to blood flow and hepatic clearance, in agreement with the literature. Certain selective patterns are seen that cannot be accounted for by vascularisation and there is some evidence to suggest higher concentrations in tumour tissue. The importance of minor metabolism and the influence of necrotic material will need to be determined before a more definite statement can be made on the advantages of 4'epiDX over its parent compound, doxorubicin.

### References

BENJAMIN, R.S., RIGGS, C.E. & BACHUR, N.R. (1977). Plasma pharmacokinetics of Adriamycin and its metabolites in humans with normal hepatic and renal function. *Cancer Res.*, **37,** 1416.

BENJAMIN, R.S., RIGGS, C.E., SERPIK, A.A. & 2 others (1974). Biliary excretion of Adriamycin (A) in man. *Clin. Res.*, **22,** 483 A.

BONFANTE, V., BONADONNA, G., VILLANI, F., DIFRONZA, G., MARTINI, A. & CASAZZA, A.M. (1979). Preliminary phase I study of 4'epi-adriamycin. *Cancer Treat. Rep.*, **63,** 915.

BONFANTE, V., BONADONNA, G., VILLANI, F. & 3 others (1980). Preliminary clinical experience with 4'epiadriamycin in advanced human neoplasia. *Recent Results Cancer Res.*, **74,** 202.

BROGGINI, M., COLOMBO, T., MARTINI, A. & DONELLI, M.G. (1980). Studies on the comparative distribution and biliary excretion of Doxorubicin and 4'Epi-doxorubicin in mice and rats. *Cancer Treat. Rep.*, **64,** 897.

CASAZZA, A.M., DI MARCO, A., BERTAZZOLI, C. & 3 others (1978). Antitumor activity, toxicity and pharmacological properties of 4'Epi-doxorubicin. *Curr. Chemother.*, **2,** 1257.

CHAN, K.K., COHEN, J.L., GROSS, J.F., HIMMELSTEIN, K.J. & BATEMAN, J.R. (1978). Prediction of adriamicin disposition in cancer patients using a physiologic pharmacokinetic model. *Cancer Treat. Rep.*, **62,** 1161.

DONELLI, M.G., BARBIERI, B., ERBA, E. & 4 others (1979). *In vitro* uptake and citotoxicity of Adriamycin in primary and metastatic Lewis lung carcinoma. *Eur. J. Cancer*, **15,** 1121.

MORO, E., JANNUSN, M.G., RANGHIERI, M. & 2 others (1981). Determination of 4'epidoxorubicin and its 13 diidroderivate in human plasma by high performance liquid chromatography with fluorescence detection. *J. Chromatogr.* (*Biomed. Appl.*) (in press).

NATALE, N., BRAMBILLA, M., LUCHINI, S. & 6 others (1981). 4'epidoxorubicin and doxorubicin: toxicity and pharmacokinetics in cancer patients. *12th International Congress of Chemotherapy—Florence* Extended Abstr., 348.

ROSSO, R., RAVAZZINI, C., ESPOSITO, M., SALA, R. & SANTI, L. (1972). Plasma and urinary levels of Adriamycin in man. *Eur. J. Cancer*, **8,** 455.